# Supplementary material for: Phylogeny and molecular evolution of the first local monkeypox virus cluster in Guangdong Province, China
Source: Nat Commun. 2023 Dec 12;14:8241. doi: 10.1038/s41467-023-44092-3 (PMC10716143; doi:10.1038/s41467-023-44092-3)
Supplement: Supplementary file 2 — Description of Additional Supplementary Files [file 41467_2023_44092_MOESM2_ESM.pdf]

Description of Additional Supplementary Files

Supplementary Data 1: Sheet 1. Epidemiological details of 10 patients infected with MPXV in Guangdong Province and their corresponding monkeypox virus genome information; **Sheet 2.** Complete genome sequences of monkeypox virus (n=1703) downloaded from GISAID database (as of September 12, 2023); **Sheet 3.** Public monkeypox virus complete genome sequences used for phylogenetic analysis; **Sheet 4.** SNPs separating the Guangdong 2023 outbreak cluster from the reference genome sequence MPXV_USA_2022_MA001; **Sheet 5.** Molecular evolution of the unique mutation spectrum of MPXV clusters with time continuity divided from the IIb C.1 lineage; **Sheet 6.** Distribution of beginning and ending breakpoints for 236 MPXV with the same recombination event.

Supplementary Data 2: Alignment used to construct phylogenetic tree in Figure 1.

Supplementary Data 3: Alignment used to construct recombination analysis.
