# Supplementary material for: Phylogeny and molecular evolution of the first local monkeypox virus cluster in Guangdong Province, China
Source: Nat Commun. 2023 Dec 12;14:8241. doi: 10.1038/s41467-023-44092-3 (PMC10716143; doi:10.1038/s41467-023-44092-3)
Supplement: Supplementary file 6 — Reporting Summary [file 41467_2023_44092_MOESM6_ESM.pdf]

nature portfolio

Corresponding author(s): Baisheng Li, Wei Zhao and Chenguang Shen

Last updated by author(s): Nov 19, 2023

Reporting Summary

Nature Portfolio wishes to improve the reproducibility of the work that we publish. This form provides structure for consistency and transparency in reporting. For further information on Nature Portfolio policies, see our Editorial Policies and the Editorial Policy Checklist.

Statistics

For all statistical analyses, confirm that the following items are present in the figure legend, table legend, main text, or Methods section.

n/a	Confirmed	
☐	☒	The exact sample size (*n*) for each experimental group/condition, given as a discrete number and unit of measurement
☐	☒	A statement on whether measurements were taken from distinct samples or whether the same sample was measured repeatedly
☐	☒	The statistical test(s) used AND whether they are one- or two-sided<br>*Only common tests should be described solely by name; describe more complex techniques in the Methods section.*
☐	☒	A description of all covariates tested
☐	☒	A description of any assumptions or corrections, such as tests of normality and adjustment for multiple comparisons
☐	☒	A full description of the statistical parameters including central tendency (e.g. means) or other basic estimates (e.g. regression coefficient) AND variation (e.g. standard deviation) or associated estimates of uncertainty (e.g. confidence intervals)
☐	☒	For null hypothesis testing, the test statistic (e.g. *F*, *t*, *r*) with confidence intervals, effect sizes, degrees of freedom and *P* value noted<br>*Give P values as exact values whenever suitable.*
☒	☐	For Bayesian analysis, information on the choice of priors and Markov chain Monte Carlo settings
☒	☐	For hierarchical and complex designs, identification of the appropriate level for tests and full reporting of outcomes
☒	☐	Estimates of effect sizes (e.g. Cohen's *d*, Pearson's *r*), indicating how they were calculated

Our web collection on statistics for biologists contains articles on many of the points above.

Software and code

Policy information about availability of computer code

Data collection	The epidemiological investigations, sample collection, and viral isolation and cultivation were conducted by Guangdong Center for Disease Control and Prevention with the informed consent of the patient, following the Guidelines for the diagnosis and treatment of mpox (2022 edition) issued by the National Health Commission of China. The use of custom code or mathematical algorithm was not involved in this study.
Data analysis	NextClade v2.14.1 (https://clades.nextstrain.org/), MAFFT v7 (https://mafft.cbrc.jp/alignment/server/add_sarscov2.html?mar15), MEGA v11 (https://www.megasoftware.net/), IQTREE v2.2.2.6 (http://www.iqtree.org/), FigTree v1.4.4 (http://tree.bio.ed.ac.uk/software/figtree/), RDP v4.101 (http://web.cbio.uct.ac.za/~darren/rdp.html) and Graphpad Prism v9.5.1 (https://www.graphpad-prism.cn/).

For manuscripts utilizing custom algorithms or software that are central to the research but not yet described in published literature, software must be made available to editors and reviewers. We strongly encourage code deposition in a community repository (e.g. GitHub). See the Nature Portfolio guidelines for submitting code & software for further information.

Data

Policy information about availability of data

All manuscripts must include a data availability statement. This statement should provide the following information, where applicable:

- Accession codes, unique identifiers, or web links for publicly available datasets
- A description of any restrictions on data availability
- For clinical datasets or third party data, please ensure that the statement adheres to our policy

All analytical data are available within Article, Figures, and Supplementary Data. A total of 10 local MPXV whole genome sequences from outbreaks in Guangdong were included in this study. The global MPXV genetic diversity sequence dataset was created based on the GISAID public database (https://www.epicov.org/epi3/frontend#290a7a), and the reference strains MPXV-M5312_HM12_Rivers (lineage IIb A, GenBank Accession No. NC_063383.1) and MPXV_USA_2022_MA001 (lineage IIb B.1, GenBank Accession No. ON563414.3) were included from the NCBI virus database (https://www.ncbi.nlm.nih.gov/labs/virus/vssi/#/virus SeqType_s=Nucleotide&Completeness_s=complete&VirusLineage_ss=taxid:10244). All ten local MPXV whole genome sequences from outbreaks in Guangdong in this study sequenced using second-generation sequencing technology on an Illumina Miniseq instrument. The MPXV DNA was extracted with the CqEx-DNA/RNA kit (Tianlong, China), amplified to 2500 kb fragment by PCR using the Monkeypox Whole Genome Sequencing kit (Cyanines, China), and sheared to approximately 500 bp fragments using the Nextera XT DNA Library Prep kit (Illumina, USA). Assembly was performed using the IPH-nano sequencing analysis software with the reference genome of strain, MPXV-M5312_HM12_Rivers (lineage IIb A, GenBank Accession No. NC_063383.1). The assembled whole genome sequences have been deposited in the GenBase in National Genomics Data Center, Beijing Institute of Genomics, Chinese Academy of Sciences/China National Center for Bioinformation, under accession number from C_AA038923.1 to C_AA038932.1 that are publicly accessible at https://ngdc.cncb.ac.cn/genbase, and the raw sequencing data have been deposited in the Genome Sequence Archive (Genomics, Proteomics & Bioinformatics 2021) in National Genomics Data Center (Nucleic Acids Res 2022), China National Center for Bioinformation / Beijing Institute of Genomics, Chinese Academy of Sciences (GSA: CRA012147) that are publicly accessible at https://ngdc.cncb.ac.cn/gsa. The detailed information has been summarized and show in Table S1.

Research involving human participants, their data, or biological material

Policy information about studies with human participants or human data. See also policy information about sex, gender (identity/presentation), and sexual orientation and race, ethnicity and racism.

Reporting on sex and gender	The 'Male' reported in Table S1 is based on the standard of 'sex', i.e., defined based on currently understood biological differences between females and males, including chromosomes, sex organs, and endogenous hormonal profiles.
Reporting on race, ethnicity, or other socially relevant groupings	These groupings are not applicable to this study
Population characteristics	The analysis of population characteristics is not applicable to this study
Recruitment	The recruitment of human participants is not involve in this study. The 10 MPXV sequences analyzed in this study were from the first 10 confirmed mpox patients reported in Guangdong Province.
Ethics oversight	This study was approved by the Ethics Committee of Guangdong Provincial Center for Disease Control and Prevention (Guangdong CDC) and complies with all relevant ethical regulations. The epidemiological investigations, sample collection, and viral isolation and cultivation were conducted by Guangdong CDC with the informed consent of the patient, following the Guidelines for the diagnosis and treatment of mpox (2022 edition) issued by the National Health Commission of China. The data analysis process is anonymous. Any information involving the patient's privacy, such as name, ID card number, telephone number, age, etc., was deleted by Guangdong CDC before analysis and was made strictly confidential to the data analyst of this study.

Note that full information on the approval of the study protocol must also be provided in the manuscript.

Field-specific reporting

Please select the one below that is the best fit for your research. If you are not sure, read the appropriate sections before making your selection.

☒ Life sciences ☐ Behavioural & social sciences ☐ Ecological, evolutionary & environmental sciences

For a reference copy of the document with all sections, see nature.com/documents/nr-reporting-summary-flat.pdf

Life sciences study design

All studies must disclose on these points even when the disclosure is negative.

Sample size	The determination of sample size was based on the Viral load (defined by the real-time PCR threshold cycle values) of the samples and their gene sequencing results. The herpes collection fluid or the isolated virus with high Viral load were used for the second-generation sequencing to assemble the MPXV whole genome sequences. The 10 MPXV sequences analyzed in our study were from the first 10 confirmed patients infected with MPXV reported in Guangdong Province and their origin have covered several cities in Guangdong Province that experienced mpox outbreaks during the same period. The goal of the final determination of the sample size for the 10 MPXV sequences was to provide the first strong report of the phylogeny and molecular evolution of the Monkeypox virus from the first local outbreak in Guangdong Province in

2023.

Data exclusions	As mentioned above, samples with low Viral load were excluded. All the samples first used for sequencing were herpes collection fluid, but due to insufficient sequencing depth and coverage, the complete MPXV genome sequence could not be assembled in the herpes collection fluid of patients M23011 and M23008. Therefore, these two MPXV sequences were sequenced and assembled from the first-generation virus isolated from herpes collection fluid.
Replication	No applicable. All the above samples were sequenced and assembled using the second-generation sequencing technique to obtain the complete genome sequence data of monkeypox. The reliability and availability of the sequence data were guaranteed by sequencing depth and coverage with the reference genome.
Randomization	No applicable. As described above, we have sequenced all available herpes collection fluid samples with high Viral load, as well as the isolated virus from M23011 and M23008.
Blinding	No applicable. Our data collection and analysis process were based on the above available MPXV sequences for phylogenetic and molecular evolution characterization, and the epidemiological investigation data involved are anonymous. Any information involving the patient's privacy, such as name, ID card number, telephone number, age, etc., was deleted by Guangdong CDC before analysis and was made strictly confidential to the data analyst of this study.

Reporting for specific materials, systems and methods

We require information from authors about some types of materials, experimental systems and methods used in many studies. Here, indicate whether each material, system or method listed is relevant to your study. If you are not sure if a list item applies to your research, read the appropriate section before selecting a response.

Materials & experimental systems

n/a	Involved in the study	
☒	☐	Antibodies
☒	☐	Eukaryotic cell lines
☒	☐	Palaeontology and archaeology
☒	☐	Animals and other organisms
☒	☐	Clinical data
☒	☐	Dual use research of concern
☒	☐	Plants

Methods

n/a	Involved in the study	
☒	☐	ChIP-seq
☒	☐	Flow cytometry
☒	☐	MRI-based neuroimaging
